# Dietary risk factors for colorectal cancer in Uganda: a case-control study

**DOI:** 10.1186/s40795-024-00894-2

**Published:** 2024-06-19

**Authors:** Richard Wismayer, Julius Kiwanuka, Henry Wabinga, Michael Odida

**Affiliations:** 1 Department of Surgery, Masaka Regional Referral Hospital, Masaka, Uganda; 2 Department of Surgery, Faculty of Health Sciences, Equator University for Science and Technology, Masaka, Uganda; 3 Department of Surgery, Faculty of Health Sciences, Habib Medical School, IUIU University, Kampala, Uganda; 4 Department of Pathology, School of Biomedical Sciences, College of Health Sciences, Makerere University, Kampala, Uganda; 5 Department of Epidemiology and Biostatistics, School of Public Health, College of Health Sciences, Makerere University, Kampala, Uganda; 6 Department of Pathology, Faculty of Medicine, Gulu University, Gulu, Uganda

**Keywords:** Colorectal cancer, Uganda, Protective factors, Risk factors, High-income developed countries, Low-income developed countries

## Abstract

**Introduction:**

Low-income countries in East Africa have a lower incidence of colorectal cancer (CRC) than high-income countries; however, the incidence has steadily increased in the last few decades. In Uganda, the extent to which genetic and environmental factors, particularly dietary factors, contribute to the aetiology of CRC is unclear. Therefore, the objective of our study was to determine the relationship between dietary factors and CRC in Uganda.

**Methods:**

We conducted a case-control study and recruited 128 cases and 256 controls, matched for age (± 5 years) and sex. Data regarding the frequency of consumption of the dietary factors were obtained from all the participants using an interview-based questionnaire. The potential dietary risk factors and protective factors evaluated included the type and frequency of meat consumed and the type and frequency of high-fibre foods consumed. The frequency was either 4 or more times/week, 2–3 times/week, once/week or never. Conditional logistic regression analyses were used to determine the odds ratios associated with the different risk and protective factors.

**Results:**

The median age (IQR) for the case participants was 55.5 (43-67.5) years, and that of the control participants was 54 (42–65) years. The male-to-female ratio was 1:1 for all the participants. Factors significantly associated with CRC cases included:- the consumption of boiled beef 2–3 times/week (aOR:3.24; 95% CI: 1.08–9.69; *p* < 0.035). Consumption of high-fibre foods, including:- millet for ≥ 4 times/week (aOR: 0.23; 95% CI: 0.09–0.62; *p* = 0.003)), spinach for ≥ 4 times/week (aOR:0.32; 95% CI: 0.11–0.97; *p* = 0.043), and potatoes 2–3 times/week (aOR: 0.30; 95% CI: 0.09–0.97; *p* = 0.044), were protective against CRC. Boiled cassava showed a tendency to reduce the likelihood of CRC when consumed ≥ 4 times/week (aOR:0.38; 95% CI: 0.12–1.18) however this did not reach statistical significance (*p* = 0.093).

**Conclusions:**

The consumption of boiled beef increases the risk of CRC, while the intake of high-fibre foods may reduce the risk of CRC among Ugandans. We recommend nutritional educational programmes to increase public awareness regarding the protective role of a high-fibre diet and to limit the intake of cooked meat in our Ugandan population.

**Supplementary Information:**

The online version contains supplementary material available at 10.1186/s40795-024-00894-2.

## Introduction

Colorectal cancer (CRC) is the sixth most common cancer in Africa, and globally is the third most common cancer [1, 2]. In Uganda, noncommunicable diseases, including CRC are on the rise. The age-standardized incidence of CRC has steadily increased from 6.8 to 11.0 per 100,000 from 1991 to 2015 [3, 4]. Despite this increase in CRC, the rate in developed high-income countries is three times higher compared to developing low-income countries [5]. Other parts of Sub-Saharan Africa including Kenya, Zimbabwe, Nigeria and Mozambique have also registered an increase in the burden of colorectal cancer [6–8]. In Uganda, the increase in the incidence of CRC may be due to an improved diagnosis, however, changes in diet and lifestyle may be responsible [9].

In developed high-income countries, research has been carried out to determine molecular abnormalities and genetic defects associated with colorectal adenomas and CRC and upscale screening programmes in the past two decades [10–13]. This has resulted in promising results in developed high-income countries with many CRC cases now diagnosed before the onset of symptoms. However, in our population, patients present with advanced-stage CRC with weight loss, altered bowel habit, abdominal discomfort, rectal bleeding and anaemia which predict a poor prognosis [14]. An important aspect of CRC prevention programmes, is knowledge of risk factors particularly dietary risk factors which is useful in the identification of high-risk individuals [15, 16].

Western literature has shown that dietary factors tend to account for the majority of sporadic colorectal cancers [17]. The International Agency for Research on Cancer (IACC) has classified a high intake of red meat as ‘carcinogenic’ to humans [18]. The increase in the incidence rate of CRC may be due to changes in the traditional Ugandan diet and the adoption of a Western diet, and these environmental factors may be responsible especially in urban areas of Uganda. Other dietary factors including animal fat, sugar and a high intake of alcohol may be associated with an increased risk of CRC [17].

A high intake of dietary fibre such as fruits, vegetables, vitamin D, calcium and milk is associated with a reduced risk of CRC [17, 18]. However, the overall importance of diet is more complex and important than the degree of risk attributed to the individual dietary components. The overall diet also has a substantial impact on the composition of gut microbiota, which plays a major role in the development of CRC [19].

In Sub-Saharan Africa, previous studies have focused on the protective role of individual dietary constituents [9]. Two studies in Uganda attempted to identify some risk factors associated with CRC however, they did not determine the dietary risk factors [20, 21]. Given the ongoing changes in dietary practices in Uganda, there is a need to evaluate the role of dietary patterns. There has been a trend towards an increased consumption of red meat and high-energy foods with a decreased consumption of high-fibre foods over the last two decades in the country [22]. With the reported increase in the incidence of noncommunicable diseases including CRC from the Kampala Cancer Registry in Uganda, knowledge of the dietary risk factors causing CRC in our population is important. Therefore, the objective of this study was to establish whether there is an association between dietary factors and colorectal cancer in Uganda.

## Methodology

### Study population

This was a hospital-based case-control study of adult black Ugandans with colorectal cancer and controls which was carried out between September 2019 to September 2021. The cases were recruited from the clinical and endoscopic services within Masaka Regional Referral Hospital, Mulago National Referral Hospital, Uganda Martyrs’ Hospital Lubaga and Mengo Hospital. Cases had a histologically proven diagnosis of colorectal adenocarcinoma [23], and were considered for inclusion if the diagnosis was within six months of the study period, to limit recall bias related to dietary factors. Cases with recurrent colorectal cancer were excluded. In all the hospitals, all cases meeting the selection criteria during the study period were included. The controls were randomly selected patients who were non-relatives to the cases and admitted for other surgical conditions in the same surgery wards. Two controls were selected for each case, and they were matched for sex and age (± 5 years of the case). The inclusion criteria for the controls included participants with a negative faecal occult blood test. As determined from their medical history and general physical examination, controls had no type of cancer. Participants not willing to provide faecal samples for faecal occult blood testing were excluded as controls. Relatives of case participants and participants testing positive for faecal occult blood were also excluded as controls. Control participants were referred for a colonoscopy to the endoscopy units in the respective hospital sites if they tested positive for faecal occult blood.

### Data collection

All participants were interviewed using a validated food frequency questionnaire after obtaining informed consent [24]. The same food-frequency questionnaire was used for case and control participants. Data on age and sex of the participants, type and frequency of meat consumed (either ≥ 4 times/week or 2–3 times/week or once/week or never) and the type and frequency of fibre consumed (either ≥ 4 times/week or 2–3 times/week or once/week or never). Smoking and BMI status was obtained from all the participants. The case and control participants were interviewed in the hospital sites.

### Statistical analysis

Continuous variables were summarized by medians (interquartile range (IQR)) while categorical variables were summarized by counts and percentages. During bivariate analysis, we used conditional logistic regression models to establish relationships between meats or high-fibre foods (variables) and CRC status. We then included meats or high-fibre foods with a p-value of ≤ 0.20 at bivariate into a multivariable model by forward selection in a stepped wedge manner. We reported variables with a p-value less than 0.05 in the multivariable analysis as independently associated with CRC status. Final multivariable models were adjusted for, by alcohol status (whether or not a respondent consumes), smoking status (whether or not a respondent smokes), residence (rural and urban) and body mass index (BMI), as such variables have been linked to differentials in CRC status in the literature (24).

### Ethical considerations

This work was part of the PhD study, which was approved by the Higher Degrees Research and Ethics Committee, School of Biomedical Sciences, College of Health Sciences, Makerere University (reference number: SBS-HDREC-630) and Uganda National Council for Science and Technology (reference number: HS-2574). Written informed consent was obtained from all participants included in the study before completing the questionnaire form. Written informed consent was obtained before obtaining a biopsy confirming colorectal adenocarcinoma on the case participants and before obtaining a faecal occult blood sample in control participants. Those control participants who tested positive for faecal occult blood were referred for screening investigations, particularly a colonoscopy, to rule out or confirm colorectal malignancy. All data pertaining to the research were kept as confidential as possible and did not identify any particular individual. The conduct of this study was in accordance with the principles outlined in the Declaration of Helsinki.

## Results

There were 128 CRC case participants and 256 control participants. The median age (IQR) for the case participants was 55.5 (43-67.5) years, and that of the control participants was 54 (42–65) years. The male: female ratio for all the participants was 1:1 (Table 1).

**Table 1:** Basic characteristics of the study participants

Characteristic	Categories	Cases (*n*)	Cases (%)	Controls (*n*)	Controls (%)
Sex	Male	63	49.2	128	50.0
	Female	65	50.8	128	50.0
Median (IQR) age		55.5 (43-67.5)		54 (42–65)	
Age Groups (years)	< 20	3	2.3	5	2.0
	20–39	20	15.6	44	17.2
	40–59	53	41.4	110	43.0
	60+	52	40.6	97	37.8
BMI (kg/m^2^)	< 25	69	53.9	192	75.0
	25-29.9	40	31.3	44	17.2
	≥ 30	19	14.8	20	7.8
Residence	Rural	10	7.8	218	85.2
	Urban	118	92.2	38	14.8
Alcohol Status	Yes	45	35.2	40	15.6
	No	83	64.8	216	84.4
Smoking Status	Yes	19	14.8	27	10.5
	No	109	85.2	229	89.5

There were 19 (14.8%) case participants and 20 (7.8%) control participants with a BMI of ≥ 30 kg/m^2^. One hundred and eighteen (92.2%) case participants compared to 38 (14.8%) control participants lived in an urban residence. Forty-five (35.2%) case participants compared to 40 (15.6%) control participants drank alcohol whilst 19 (14.8%) case participants and 27 (10.5%) control participants smoked cigarettes (Table 1).

Table 2 shows the crude odds ratios for the relationship between CRC status and types of meat, while Table 3 shows the crude odds ratios for the relationship between CRC status and high-fibre foods.

The following observations were made on the type and frequency of meat consumed in the bivariate analysis. Compared to individuals who never consumed roast chicken, those who consumed roast chicken once/week and 2–3 times/week were 1.88 (cOR: 1.88; *p* = 0.008) and 1.95 (cOR: 1.95; *p* = 0.073) times likely to have CRC respectively. Individuals who consumed fried chicken 2–3 times a week were 3.10 (cOR: 3.10; *p* = 0.003) times likely to have CRC compared to those who never consumed fried chicken. Compared to individuals who never consumed boiled chicken, those who consumed boiled chicken once and 2–3 times a week were 1.68 (cOR: 1.68; *p* = 0.025) and 2.31 (cOR: 2.31; *p* = 0.054) times likely to have CRC respectively (Table 2).

Consumption of boiled beef at a frequency of 2–3 times a week (2.98 (cOR:2.98; *p* = 0.001) and ≥ 4 times per week (2.15 (2.15 (cOR: 2.15; *p* = 0.078) were associated with a higher likelihood of CRC compared to no consumption of boiled beef. Consumption of roasted lamb, fried pork, and roasted pork were not associated with an increased likelihood of having CRC (Table 2).

**Table 2:** Relationship between the consumption of different types of meats and CRC status

Characteristic(s)	Categories	cOR	95% CI		*p*-value*
Roast Chicken	Never	**1.00**			
	1x a week	**1.88**	**1.18**	**3.01**	**0.008**
	2-3x a week	**1.95**	**0.94**	**4.04**	**0.073**
	4 + x a week	**-**	**-**	**-**	**-**
Fried Chicken	Never	**1.00**			
	1x a week	**1.42**	**0.89**	**2.26**	**0.142**
	2-3x a week	**3.10**	**1.48**	**6.47**	**0.003**
	4 + x a week	**0.51**	**0.06**	**4.37**	**0.538**
Boiled Chicken	Never	**1.00**			
	1x a week	**1.68**	**1.07**	**2.64**	**0.025**
	2-3x a week	**2.31**	**0.99**	**5.41**	**0.054**
	4 + x a week	**-**	**-**	**-**	**-**
Boiled beef	Never	**1.00**			
	1x a week	**0.96**	**0.51**	**1.79**	**0.889**
	2-3x a week	**2.98**	**1.58**	**5.59**	**0.001**
	4 + x a week	**2.15**	**0.92**	**5.02**	**0.078**
Roast Lamb	Never	**1.00**			
	1x a week	**0.77**	**0.33**	**1.78**	**0.537**
	2-3x a week	**2.08**	**0.49**	**8.85**	**0.319**
	4 + x a week	**4.65**	**0.41**	**53.26**	**0.217**
Fried Pork	Never	**1.00**			
	1x a week	**1.27**	**0.79**	**2.03**	**0.317**
	2-3x a week	**1.12**	**0.51**	**2.46**	**0.783**
	4 + x a week	**0.72**	**0.14**	**3.59**	**0.688**
Roast Pork	Never	**1.00**			
	1x a week	**1.06**	**0.59**	**1.90**	**0.846**
	2-3x a week	**1.33**	**0.49**	**3.55**	**0.576**
	4 + x a week	**1.55**	**0.34**	**6.94**	**0.570**

*p-values obtained using a conditional logistic regression model

**Table 3:** Relationship between the different types of high fibre foods and CRC status

Characteristic(s)	Categories	cOR	95% CI		*p*-value*
Cassava	Never	1.00			
	1x a week	1.11	0.55	2.24	0.767
	2-3x a week	1.10	0.55	2.22	0.779
	4 + x a week	0.53	0.27	1.01	0.052
Millet	Never	1.00			
	1x a week	0.62	0.34	1.12	0.114
	2-3x a week	0.65	0.34	1.24	0.191
	4 + x a week	0.39	0.21	0.70	0.002
Beans	Never	1.00			
	1x a week	0.44	0.14	1.33	0.146
	2-3x a week	0.36	0.12	1.03	0.057
	4 + x a week	0.44	0.16	1.22	0.117
Rice	Never	1.00			
	1x a week	1.03	0.50	2.13	0.930
	2-3x a week	0.79	0.38	1.66	0.534
	4 + x a week	0.46	0.22	0.93	0.032
Maize	Never	1.00			
	1x a week	0.82	0.42	1.61	0.567
	2-3x a week	0.70	0.35	1.39	0.303
	4 + x a week	0.74	0.37	1.47	0.390
Matooke	Never	1.00			
	1x a week	0.82	0.32	2.10	0.676
	2-3x a week	0.71	0.31	1.63	0.419
	4 + x a week	0.90	0.46	1.75	0.758
Sorghum	Never	1.00			
	1x a week	0.76	0.36	1.60	0.470
	2-3x a week	1.46	0.63	3.33	0.375
	4 + x a week	0.46	0.13	1.62	0.228
Cabbage	Never	1.00			
	1x a week	0.72	0.40	1.27	0.255
	2-3x a week	0.61	0.48	1.75	0.799
	4 + x a week	0.59	0.29	1.21	0.151
Potatoes	Never	1.00			
	1x a week	1.03	0.54	1.98	0.919
	2-3x a week	0.61	0.32	1.15	0.129
	4 + x a week	0.66	0.34	1.28	0.221
Spinach	Never	1.00			
	1x a week	0.69	0.36	1.31	0.254
	2-3x a week	0.63	0.35	1.13	0.122
	4 + x a week	0.60	0.31	1.19	0.146
Green Peppers	Never	1.00			
	1x a week	1.01	0.55	1.83	0.983
	2-3x a week	0.73	0.40	1.35	0.318
	4 + x a week	0.76	0.43	1.32	0.324
Banana	Never	1.00			
	1x a week	0.40	0.15	1.03	0.058
	2-3x a week	0.42	0.17	1.07	0.069
	4 + x a week	0.38	0.15	0.93	0.034
Watermelon	Never	1.00			
	1x a week	0.61	0.36	1.06	0.077
	2-3x a week	0.76	0.42	1.36	0.348
	4 + x a week	0.84	0.43	1.64	0.613
Oranges	Never	1.00			
	1x a week	0.84	0.52	1.36	0.489
	2-3x a week	0.92	0.46	1.87	0.824
	4 + x a week	0.75	0.38	1.48	0.412
Mangoes	Never	1.00			
	1x a week	0.67	0.36	1.22	0.187
	2-3x a week	0.67	0.35	1.30	0.234
	4 + x a week	0.62	0.31	1.26	0.186

*p-values obtained using a conditional logistic regression model

Table 3 shows the observations which were made on the type and frequency of high-fibre foods consumed in the bivariate analysis. Compared to individuals who never consumed cassava, those who consumed ≥ 4 times/week were less likely to be cases of CRC (cOR: 0.53; *p* = 0.052), although statistical significance was borderline. Consumption of millet at ≥ 4 times/week (cOR: 0.39; *p* = 0.002) was associated with a less likelihood of being a CRC case. Similarly, consumption of rice for ≥ 4 times/week (cOR: 0.46; *p* = 0.032) and bananas for ≥ 4 times/week (cOR: 0.38; *p* = 0.034) were associated with a lesser likelihood of being a CRC case.

The other high-fibre foods, in particular beans, maize, matooke, sorghum, cabbage, potatoes, spinach, green peppers, watermelon, oranges and mangoes, also tended to have a protective effect against CRC but did not reach statistical significance (Table 3).

**Table 4:** Factors associated with CRC status in the multivariable model

Characteristic(s)	Categories	aOR	95% CI		*p*-value^*^
Boiled beef	Never	1			
	1x a week	1.70	0.61	4.78	0.311
	2-3x a week	3.24	1.08	9.69	0.035
	4 + x a week	1.73	0.45	6.72	0.428
Fried Chicken	Never	1.00			
	1x a week	0.94	0.41	2.12	0.877
	2-3x a week	0.84	0.25	2.84	0.774
	4 + x a week	0.28	0.02	5.24	0.395
Rice	Never	1.00			
	1x a week	1.56	0.45	5.32	0.482
	2-3x a week	1.14	0.32	4.00	0.838
	4 + x a week	0.67	0.20	2.21	0.513
Cassava	Never	1.00			
	1x a week	0.76	0.24	2.38	0.643
	2-3x a week	0.85	0.26	2.78	0.79
	4 + x a week	0.38	0.12	1.18	0.093
Millet	Never	1.00			
	1x a week	0.46	0.17	1.24	0.125
	2-3x a week	0.53	0.18	1.54	0.242
	4 + x a week	0.23	0.09	0.62	0.003
Spinach	Never	1.00			
	1x a week	0.74	0.27	2.07	0.57
	2-3x a week	0.61	0.21	1.73	0.349
	4 + x a week	0.32	0.11	0.97	0.043
Potatoes	Never	1.00			
	1x a week	0.55	0.17	1.80	0.321
	2-3x a week	0.30	0.09	0.97	0.044
	4 + x a week	0.70	0.22	2.23	0.542

*p-values obtained using an ordinary logistic regression model

In the multivariable analysis, the following findings were observed. Boiled beef was associated with an increased likelihood of being a CRC especially when consumed at a frequency of 2–3 times/week (aOR: 3.24; *p* = 0.035) (Table 4). The following high-fibre foods had a protective effect against CRC when eaten at a frequency of ≥ 4 times/week: millet (aOR:0.23; *p* = 0.003) and spinach (aOR: 0.32; *p* = 0.043). Consumption of potatoes at a frequency of 2–3 times/week (aOR: 0.30; *p* = 0.044) had a protective effect against CRC (Table 4).

## Discussion

Our results showed that the risk of developing CRC was inversely associated with the intake of millet and spinach. The intake of red meat, particularly boiled beef was associated with an increased risk for the development of CRC. Previous epidemiological studies have supported the inverse relationship to the development of CRC from the consumption of food rich in dietary fibre such as spinach [24–29]. Matooke, cabbage, beans, mangoes, oranges and bananas, which are other high-fibre foods, were also protective against CRC, however, these effects did not reach statistical significance in our study.

Dietary fibre results in the promotion of the excretion of bile acids into the colon and the prevention of the reabsorption of bile acids. It also prevents the conversion of primary to secondary bile acids by binding to bile acids. The fermentation of fibre by colon bacteria results in the production of short-chain fatty acids. These short-chain fatty acids inhibit tumour development by causing apoptosis. Dietary fibre reduces any interaction between the colonic mucosa and the faecal mutagens by increasing the faecal bulk and reducing inflammatory markers, nitric oxide and insulin resistance [30]. Green vegetables provide dietary fibre which tends to be protective against CRC [31, 32]. The type of fibre consumed determines the protective effect, with cereal fibre having a low protective effect and vegetable and fruit having a high protective effect [31, 32]. The possible mechanisms for dietary fibre protecting against CRC include: (i) a reduced colonic transit time; (ii) the formation of short-chain fatty acids in the colon such as acetate, butyrate and propionate from the fermentation of dietary fibre; (iii) Fruits contain folic acid which reduces the risk of CRC; (iv) Protection against oxidative tissue damage by selenium from cereals which acts as a cofactor for glutathione oxidase; (v) Resistant starch and indigestible oligosaccharides reducing the ability of bile acids to act as carcinogens; (vi) anticarcinogenic compounds in vegetables and fruits, including organosulfides, carotenoids, vitamin C, isothiocyanates, flavonoids, resveratrol and protease inhibitors [33]. Our study has shown that food rich in dietary fibre such as spinach was protective against CRC. These findings are consistent with those from other studies which have shown a protective role against CRC in vegetable and fruit fibre, compared to cereal fibre [34–38].

In Uganda, vegetables form part of the traditional diet and are consumed by rural populations due to their accessibility, availability and affordability. They include cruciferous vegetables such as cabbage and dark green leafy vegetables such as cassava leaves and spinach. Our study showed that the consumption of spinach and cassava leaves is associated with a reduced risk of CRC. These vegetables are a rich source of ascorbic acid, folate, retinol, minerals such as iron and magnesium and have a high content of dietary fibre [39]. Folates stabilize tumour suppressor genes and suppress tumour cell proliferation, while retinol and ascorbic acid have antioxidant properties and are antitumorigenic [40]. These vegetables have the following phytochemicals which include phenolic compounds, alkaloids, terpenoids and flavonoids, which inhibit reactive oxygen species and hence are cytotoxic, and prevent alteration of DNA. Experimental studies have also shown that induction of metabolism of 2-amino-1-methyl-6-phenylimidazo [4–6] pyridine from the consumption of cruciferous vegetables reduces CRC [32].

In Uganda, tubers (potato, cassava), cereals (rice, millet, sorghum) and bananas (matooke, green bananas) are the main staple foods. These staple foods have substances with anticarcinogenic properties such as polyphenols, minerals and vitamins. A high content of non-digestible carbohydrates (NDCs) such as fibre and resistant starch are found in roots and tubers [41]. Our study showed that consumption of potatoes at a frequency of two to three times/weekly was associated with a reduced likelihood of CRC. These staple crops contain starch and nonstarch polysaccharides which are anaerobically fermented to short-chain fatty acids such as butyrate, acetate and propionate [41]. The production of butyrate from the fermentation of resistant starch is more protective against CRC than the nonstarch polysaccharides of dietary fibre [32, 41–46].

The low pH in the colonic lumen, favours the production of short-chain fatty acids from gut microbiota, which bind to carcinogens in the colon, alter preneoplastic lesions and suppress mutations [47]. An improvement in host immunity through the regulation of T-helper cells, B-cells, cytotoxic cells and suppression of inflammation results from the interaction of colonocytes with short-chain fatty acids [48]. A high intake of nondigestible carbohydrates from these staple foods has resulted in a 40% reduction in the risk of CRC in another study [49]. In our study, cassava tended to show a reduced tendency for CRC however this did not reach statistical significance. Cassava is protective against CRC as it contains tamarin, which is a chemical responsible for producing hydrocyanide. In vitro experimental studies, have found that the toxicity from hydrocyanide causes the death of CRC cancer cells [50].

Previous in vitro and in vivo studies have shown that foxtail millet bran (FMBP) produces a secretory peroxidase against CRC [51]. Foxtail millet bran (FMBP) targets cell surface glucose-regulated protein 78 (csGRP78) which is abnormally located on CRC. FMBP also acts against the nucleotide-binding domain (NBD) of csGRP78 which interferes with the activation of STAT3 (signal transducer and activator of transcription 3) in CRC cells and results in the accumulation of reactive oxygen species and hence CRC cell growth inhibition [51]. These findings are consistent with the results of our study which showed that millet was also protective against CRC.

Maize was found to be protective against CRC however, this did not reach statistical significance in our study. Studies have shown that compared to potato starch, resistant starch from maize, produces more butyrate, which is preferable for colonocytes [42]. Our findings showed that the consumption of potatoes 2-3x/weekly, was protective against CRC, as it is a high-fibre food which produces short-chain fatty acids, particularly butyrate which is preferable for colonocytes and this high-fibre food also produces antioxidants [41]. The Ugandan diet tends to consist of a variety of potatoes, millet-based meals, maize and cassava, and hence, these high-fibre foods tend to be protective against CRC.

In Uganda, legumes, particularly beans, are an important source of protein in the diet. Phytoestrogens are anti-carcinogenic and are contained in beans. Genistein, a phytoestrogen, mediates a cancer promotor EGF protein, which inhibits the proliferation of HT-29 colon cancer cells [52]. Flavonoids tend to be anti-carcinogenic as they have a high folate content and cause apoptosis of colon cancer cells. The findings from our study showed that beans are protective against CRC, however, this did not reach statistical significance. A high consumption of legumes in Asians has been found to reduce the development of CRC [53]. Meat is high in protein and is associated with CRC, therefore, substituting this food with legumes will potentially reduce the development of CRC, particularly in urban parts of Uganda.

The traditional Ugandan diet includes regular fruits such as bananas, mango and watermelon and vegetable fruits such as green peppers. Phytochemicals such as flavonoids and phenols, vitamins A, B,C, D,E and minerals are present in these fruits. Tocopherol and retinol reduce epithelial cell proliferation and decrease the toxic effects of reactive oxygen species in the causation of colon cancer. Ascorbic acid prevents tumour progression and has chemosensitizing properties against CRC cells [54]. The minerals magnesium, phosphorous, zinc and selenium increase the expression of antioxidant enzymes and have a protective role against CRC [54].

In Uganda, the stew base includes green peppers which are rich in antioxidants [40]. High-income developed countries have a high rate of CRC due to a low consumption of dietary fibre and a high consumption of processed meat and fat [55–57]. The incidence of CRC in American blacks from migration studies is comparable to that in Caucasians. Therefore, environmental dependence on the type of diet consumed plays an important role in the development of CRC. Our urban population tends to eat cooked meat more frequently in contrast to the rural population where the consumption of meat is low due to the high cost. There is an increased risk of CRC when meat is cooked to high temperatures. Carcinogens known as heterocyclic amines are released when meat is cooked for a long period at high temperatures above 1800^0^C [55–57].

In Uganda, due to a poor electricity supply, proper refrigeration of meat is not possible. Therefore, many Ugandans deep fry meat in used oil for consumption. Meat that is grilled or barbecued releases high amounts of polycyclic aromatic hydrocarbons [57–59]. This is due to pyrolysis of fat that falls on the heat source forming smoke [60]. The major pyrolysis mutagens which are found in high heat over cooked beef include pyridoindole, quino xalines and pyridoimidazole [36]. Well-done meat contains high amounts of heterocyclic amines (HCAs) and studies from North America have found that these heterocyclic amines (HCAs) are carcinogenic and cause CRC [57]. Studies from West Africa have also found that charcoal-roasted meat has carcinogenic properties [61, 62]. However, consumption of meat in Uganda, constitutes a small part of the rural diet, resulting in minimal exposure to PAHs and HCAs. Therefore, the exposure to these carcinogenic substances is not to the magnitude at which Caucasians tend to be exposed. This difference in diet, compared to the developed western world may explain the lower incidence of colorectal carcinoma in Uganda.

In our study, the consumption of red meat was higher among the cases than the controls, Hence a positive relation between red meat consumption and the development of CRC, particularly for boiled beef. These findings are consistent with those in a systematic review by Santorelli et al., which determined the association between the consumption of meat and CRC [60]. A study by Abdulbari et al., found that 20.9% of cases had a daily consumption of meat which was higher than the 17.1% in the controls [63]. Consumption of 250 g/day of meat was reported to be associated with an increased risk of CRC by the World Cancer Research Fund International [64, 65]. There was no association found between fried pork, fried chicken and roast pork with the risk of CRC. The findings from our study may be in contrast with a study by Ahmed FE et al., which found that red meat is not a risk factor for CRC [66].

## Conclusions

This study suggests that the consumption of high-fibre foods such as millet and spinach and a moderate consumption of potatoes may reduce the risk of CRC, whilst intake of boiled red meat increases the risk of CRC. Given these findings, we recommend a nutrition education programme to limit the intake of cooked meat and to increase public awareness regarding the protective role of a high-fibre diet. In Uganda, education programmes on health promotion and nutrition at primary health centres should be considered and consumption of vegetables and fruits should be encouraged and replace foods low in fibre such as meat. Targeted CRC screening of patients with average risk factors for the disease should be considered in our population. Dietary modification will have a significant impact on reducing the development of CRC in Uganda.

### Study limitations

Case-control studies tend to be prone to selection bias [67]. To minimize this bias, controls were recruited from the surgery departments of four different specialized hospitals with diseases that had no association with dietary factors. The cases and controls recruited from private hospitals may have had a higher socioeconomic status compared to those recruited from government hospitals; however, studies have shown an inverse association between the risk of CRC and the consumption of vegetables and fruits among individuals of different socioeconomic status [68]. Similar results from controls recruited from different hospital sources suggest that their selection did not affect the results [68].

The probability of selection bias was reduced in our study due to the high participation rate for both cases (98%) and hospital-derived controls (95%). Participants may recall dietary practices differently and recall bias tends to be a concern in case-control studies [69]. The controls may have recalled their dietary consumption differently from cases as controls did not have malignant disease. If colorectal cancer patients were aware of their diagnosis, this may have led them to consciously change their well-being and health. Therefore, cases were interviewed very soon following diagnosis to reduce bias. To improve the comparability of recall between controls and cases, a standardized interview questionnaire method was used. Cognitively, it may be difficult to answer the usual frequency of consumption questions [69]. Therefore, multiple recalls were obtained for all the participants to obtain a reliable estimate of the usual intake. An adjustment for many potential confounders was made, in particular for alcohol status, smoking status, residence and body mass index (BMI); however, residual confounding bias may have occurred due to poorly measured or unmeasured variables.

Information bias may be a possible reason, which may have resulted from misclassification or recall bias which are limitations of case-control studies [70]. Nondifferential misclassification may result from a cultural context whereby some cases might have stated that they do not eat lamb or pork when they do eat these types of meat. Alternatively, without distinctively classifying the different types of meat, participants might have been likely to indicate eating meat in general terms. All the meats would have been clamped together as beef, further amplifying the misclassification of the exposure to different meats.

The interview-based questionnaire was a food frequency questionnaire which was prone to measurement error [71]. Random errors and systematic errors are the two types of measurements errors that occur from food-frequency questionnaires [71]. Systematic errors relate to the design and conduct of the study. Systematically, there was bias arising out of recall, misclassification of exposures again resulting from recall but also measurement. Random errors are those that arise out of chance and relate to the fact that sampling rather than the whole population is considered. The bigger the sample size, the smaller is the random error. In our study, the sample size was adequate and therefore random error was minimal [71]. While implementing the FFQ questionnaire in our study there was a limitation regarding the number of foods we asked for and details about food preparation. Furthermore, the recall for the consumption of various foods during the past 12 months would make it practically impossible to ascertain the quantities of foods, and or consumption of certain foods over this long period. Consuming sufficient and not excessive, essential nutrients, sources of energy and dietary fibre and not contaminants and toxins is necessary to attain optimal nutritional status [72]. Dietary intake could possibly have been inadequate in some of our study participants. Although the type of food and frequency of consumption have been addressed, the quantity of food consumed and nutrient consumption was not considered in this study.

Apart from the type of meat consumed, the processing method used will produce different levels of risk [73]. In Uganda, many foods are prepared at very high temperatures which are associated with an increased risk of CRC, compared to many parts of Europe where the cooking of meat is based on consumers’ preference as either half-done or well-done [73]. Whilst this study found an increased risk of CRC with boiled beef and fried chicken it did not evaluate the effect of fried or grilled beef on CRC. Apart from chicken, the effects of the different cooking modalities for the other types of meat were not evaluated.

Studies have shown that a high intake of red meat and a positive energetic balance from a high intake of carbohydrates and total fat is associated with a significant increase in the risk of CRC [74, 75]. A positive synergic effect has also been found between high energy intake, physical inactivity and obesity and the incidence of CRC in other studies [74, 75]. However, in this study, another limitation is that energy intake from fat and carbohydrate consumption was not assessed and therefore we were unable to adjust for these key factors.

### Electronic supplementary material

Below is the link to the electronic supplementary material.

Supplementary Material 1

## Data Availability

The data supporting the findings of this study are available from the corresponding author upon reasonable request.

## Abbreviations

CRC: colorectal cancer
EGF: epidermal growth factor
FMBP: foxtail millet bran
FFQ: food frequency questionnaire
HCA: heterocyclic amines
NBD: nucleotide-binding domain
NCD: non-digestible carbohydrates
